# Longitudinal changes in the ganglion cell complex thickness in acute central serous chorioretinopathy using spectral-domain optical coherence tomography

**DOI:** 10.1038/s41598-023-50953-0

**Published:** 2024-01-08

**Authors:** Dong Ik Kim, Ki Woong Bae, Kyuhwan Jang, Daniel Duck-Jin Hwang

**Affiliations:** 1 Department of Ophthalmology, Hangil Eye Hospital, 35 Bupyeong-daero, Bupyeong-gu, Incheon, 21388 South Korea; 2 Department of Ophthalmology, Catholic Kwandong University College of Medicine, Incheon, South Korea; 3 Department of Ophthalmology, Nowon Eulji Medical Center, Seoul, South Korea; 4 Department of Ophthalmology, Eulji University College of Medicine, Seoul, South Korea

**Keywords:** Optic nerve diseases, Retinal diseases

## Abstract

In this retrospective longitudinal cohort study, we investigated the temporal changes in the peripapillary retinal nerve fiber layer (pRNFL) and inner retinal thickness in patients with acute central serous chorioretinopathy (CSC) using spectral-domain optical coherence tomography (SD-OCT). We followed up with these patients for 6 months, and during this period, the thickness of the pRNFL and the ganglion cell complex (GCC) in CSC patients were compared with the eyes of normal healthy individuals. The study also examined the correlation between the pRNFL thickness, GCC thickness, and visual acuity. The research sample consisted of 67 patients (43 male and 24 female) with an average age of 49.72 ± 9.87 years. The initial findings showed no significant differences in the pRNFL and GCC thickness between the study and fellow eye, study and normal healthy eyes, and fellow and normal healthy eyes. There was no significant difference in the pRNFL and GCC thickness when comparing the study eye with the fellow eye for 6 months. In the study eye, no significant difference was observed when comparing the initial GCC and pRNFL thickness with those at 1, 3, and 6 months. Visual acuity improved significantly from 0.18 ± 0.23 logMAR to 0.04 ± 0.06 logMAR (*p* < 0.001). The GCC and pRNFL thickness did not significantly affect visual acuity. In conclusion, acute CSC patients did not show significant changes in the pRNFL and inner retinal thickness, suggesting that the GCC and pRNFL do not substantially influence the short-term visual prognosis in these patients.

## Introduction

Central serous chorioretinopathy (CSC), which is a disease characterized by the development of focal neuroretinal detachment, is reportedly associated with changes in the retinal pigment epithelium, hyperpermeability, and changes in the choroid^1,2^. With the recent development of optical coherence tomography (OCT) machines, quantitative analysis has become possible; thus, more information concerning the diagnosis and course of diseases can be obtained. Since CSC is classified as a pachychoroid spectrum disease, most studies to date have focused on the outer retina and choroid^3–5^, and relatively few studies on the inner retina have been conducted.

The ganglion cell complex (GCC) is a complex of three layers of the inner retina, i.e., the retinal nerve fiber layer (RNFL), ganglion cell layer (GCL), and inner plexiform layers (IPL), each of which is located in the axon, cell body, and dendrite of the ganglion cell, respectively. The importance of GCC in glaucoma and optic nerve diseases has been demonstrated in many studies^6,7^; moreover, GCC abnormality reportedly occurs in inflammatory, ischemic diseases, or retinal degeneration^8–12^.

A previous study demonstrated that the GCL and macular RNFL (mRNFL) decreased as the subretinal fluid (SRF) decreased in acute CSC^4^; moreover, another study reported that the GCC decreased compared to normal^13^ or fellow eyes^14^ when SRF was present in acute CSC. Peripapillary RNFL (pRNFL) is reportedly thicker in eyes with acute CSC compared to normal eyes^15^.

However, in previous studies, the number of patients included in the study was relatively small, and the OCT equipment used for diagnosis varied from study to study (Swept-source-OCT vs. spectral domain (SD)-OCT). Moreover, owing to the nature of the SD-OCT device used in the studies, correction for segmentation errors could not be performed^13–15^.

Therefore, in this study, affected (study) eyes of patients with acute CSC were compared with normal and fellow eyes to determine the difference in the pRNFL, GCC (mRNFL, GCL, and IPL) values and variations over time in these values. We also analyzed the relationship between these values and visual acuity in patients with CSC. Through these, this study sought to determine changes in the GCC in cases of acute CSC and investigate their potential impact on visual acuity.

## Results

A total of 67 patients were included in the study. Their average age was 49.72 ± 9.87 years; there were 43 males and 24 females. No significant differences were observed in the gender, age, and laterality between the study group and normal control group (Table 1).

**Table 1:** Patient demographics and characteristics.

	Study group	Controls	p
Patients (n)	67	60	
Age (years)	49.72 ± 9.87	48.53 ± 12.52	0.553^a^
Sex			0.572^b^
Male	43	42	
Female	24	18	
Affected eye			0.476^b^
OD	34	35	
OS	33	25	
BCVA (logMAR)	0.18 ± 0.23	0.00	< 0.001﻿^a^
IOP (mmHg)	16.3 ± 2.7	16.0 ± 2.8	0.523﻿^a^
CMT (µm)	446.99 ± 114.18	272.17 ± 24.29	< 0.001^a^

Values are presented as n or mean ± standard deviation.

*OD* right eye, *OS* left eye, *BCVA* best-corrected visual acuity, *IOP* intraocular pressure, *CMT* central macula thickness.

^a^*p*-value derived from the independent t-test.

^b^*p*-value derived from the Pearson’s Chi-square test.

### Changes in the best-corrected visual acuity, intraocular pressure (IOP), and CMT

Average visual acuity at the first visit was 0.18 ± 0.23 logMAR, and final visual acuity was 0.04 ± 0.06 logMAR, demonstrating a significant improvement (*p* < 0.001). The IOP was 16.3 ± 2.7 mmHg at the first visit and 16.0 ± 3.0 at 6 months, showing no significant difference (*p* = 0.240). CMT decreased significantly from 446.99 ± 114.18 µm initially to 251.42 ± 22.19 µm at 6 months (*p* < 0.001).

### Changes in the GCC thickness (mRNFL, GCL, and IPL)

**Figure 1:**
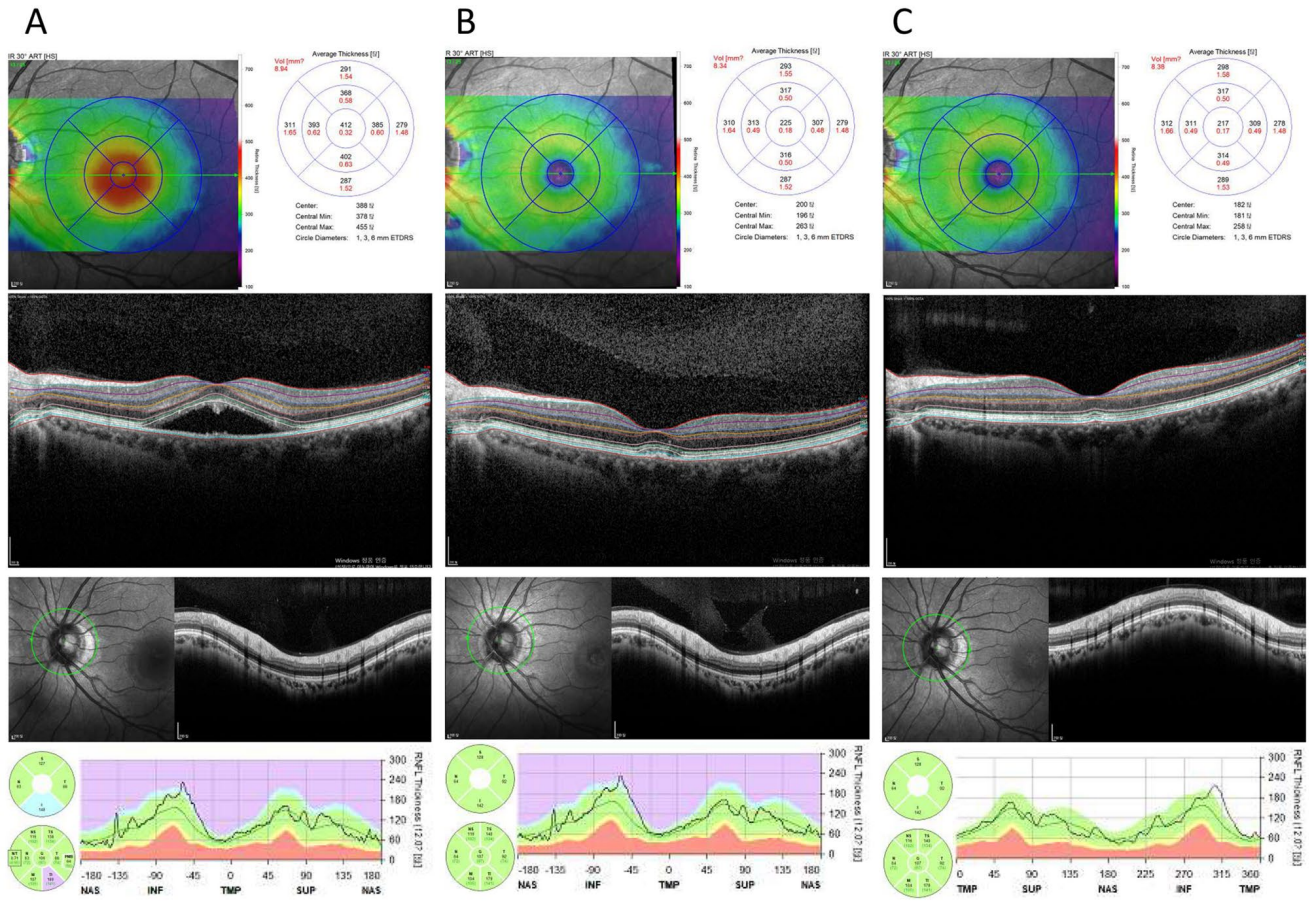
Representative case of a 53-year-old female patient. Changes in the inner retina and peripapillary retinal nerve fiber layer over time. With the gradual disappearance of subretinal fluid over time, the central macular thickness decreased; however, the thickness of the inner retina and peripapillary retinal nerve fiber layer thickness remained unchanged. (**A**) First visit, (**B**) 3-months follow up, (**C**) 6-months follow up.

At the initial visit, no significant differences were observed in the pRNFL and mRNFL values in any section upon comparing CSC eyes to fellow eyes, CSC eyes to normal controls, and fellow eyes to normal controls (Table 2). In the CSC eyes, no significant difference was observed between initial, 1, 3, and 6 month values of GCC (Table 3). Upon comparing the mRNFL, GCL, and IPL values between the baseline and follow-up for each value, no significant differences were observed at any time point from the first visit to the 6-months follow up visit (Fig. 1).

**Table 2:** Comparison of the initial peripapillary retinal nerve fiber layer thickness and inner retinal layer thickness between the central serous chorioretinopathy, fellow, and normal control eyes.

	CSC eye	Fellow eye	*p*^a^	Control eye	*p*^b^	*p*^c^
pRNFL						
G pRNFL	103.97 ± 9.46	102.32 ± 8.99	0.076	103.13 ± 10.02	0.803	0.682
TS pRNFL	141.16 ± 16.31	143.32 ± 16.53	0.383	145.87 ± 13.41	0.216	0.433
NS pRNFL	116.73 ± 27.04	112.24 ± 17.23	0.229	118.78 ± 19.16	0.573	0.086
N pRNFL	71.76 ± 10.49	69.97 ± 12.41	0.256	69.97 ± 12.13	0.362	0.697
NI pRNFL	116.78 ± 23.59	114.51 ± 22.02	0.133	108.98 ± 18.85	0.081	0.209
TI pRNFL	157.30 ± 16.67	156.73 ± 15.75	0.836	152.28 ± 19.30	0.258	0.219
T pRNFL	77.41 ± 14.75	77.32 ± 11.51	0.974	81.42 ± 12.21	0.119	0.101
GCC						
GCC	104.19 ± 7.51	104.38 ± 7.49	0.647	105.87 ± 6.71	0.301	0.291
mRNFL	33.33 ± 3.55	33.09 ± 3.13	0.276	34.10 ± 3.63	0.163	0.083
GCL	39.02 ± 3.36	39.16 ± 3.31	0.487	39.60 ± 2.54	0.547	0.221
IPL	31.84 ± 2.19	32.12 ± 2.62	0.198	32.17 ± 2.20	0.777	0.379

Values are presented as n or mean ± standard deviation.

*CSC* central serous chorioretinopathy, *G* global, *TS* superotemporal, *NS* superonasal, *N* nasal, *NI* inferonasal, *TI* inferotemporal, *T* temporal, *pRNFL* peripapillary retinal nerve fiber layer, *GCC* ganglion cell complex, *mRNFL* macular retinal nerve fiber layer, *NFL* nerve fiber layer, *GCL* ganglion cell layer, *IPL* inner plexiform layer.

^a^Comparison between the CSC and fellows eyes at initial visit. *p* value derived from the paired t-test.

^b^Comparison between the CSC and normal control eyes at initial visit. *p* value derived from the independent samples t-test.

^c^Comparison between the fellow and normal control eyes at initial follow up. *p* value derived from the independent samples t-test.

**Table 3:** Longitudinal changes in the ganglion cell complex thickness in central serous chorioretinopathy eye.

	Baseline	1 month	3 months	6 months
GCC	104.56 ± 6.69	104.33 ± 6.60	104.67 ± 7.09	104.72 ± 6.73
*p*-value^a^		0.463	0.727	0.609
mRNFL	33.35 ± 2.97	32.94 ± 2.95	33.14 ± 3.31	33.22 ± 3.05
*p*-value^a^		0.461	0.081	0.587
GCL	39.40 ± 3.16	39.24 ± 2.98	39.23 ± 3.08	39.14 ± 2.86
*p*-value^a^		0.581	0.337	0.194
IPL	32.13 ± 2.28	32.05 ± 2.18	32.34 ± 2.28	32.35 ± 2.34
*p*-value^a^		0.600	0.240	0.208

Values are presented as the mean ± standard deviation, unless otherwise indicated.

*GCC* ganglion cell complex, *mRNFL* macular retinal nerve fiber layer, *GCL* ganglion cell layer, *IPL* inner plexiform layer.

^a^Comparison between the baseline values and values at each time point. *p* value derived from the paired t-test.

### Changes in the pRNFL thickness and correlation with the mRNFL thickness

Upon comparing the CSC and fellow eyes, the pRNFL values did not demonstrate a significant difference at any time point from the first visit to the 6 month follow up visit. No significant difference was observed between the pRNFL values in the CSC eyes at the first visit and those at 1, 3, and 6 months (Table 4). No significant correlation was found between the global pRNFL values at the first and 6 months and the mRNFL values at the first and 6 months (all *p* > 0.05).

**Table 4:** Longitudinal changes in the peripapillary retinal nerve fiber layer thickness in the central serous chorioretinopathy eye.

	Baseline	1 month	3 months	6 months
G pRNFL	103.75 ± 10.11	101.83 ± 10.90	103.76 ± 10.34	102.80 ± 9.83
*p*-value^a^		0.103	0.509	0.415
TS pRNFL	139.90 ± 13.48	139.92 ± 17.32	142.35 ± 14.30	141.30 ± 15.93
*p*-value^a^		0.629	0.750	0.132
NS pRNFL	116.70 ± 26.11	116.17 ± 31.33	116.88 ± 28.39	116.65 ± 25.83
*p*-value^a^		0.742	0.479	0.975
N pRNFL	70.60 ± 10.11	71.83 ± 11.73	70.65 ± 12.84	71.30 ± 13.18
*p*-value^a^		0.927	0.912	0.604
NI pRNFL	113.80 ± 25.00	117.17 ± 30.59	111.59 ± 25.46	114.05 ± 27.97
*p*-value^a^		0.611	0.338	0.836
TI pRNFL	159.35 ± 17.18	149.92 ± 12.71	157.29 ± 15.17	154.00 ± 13.16
*p*-value^a^		0.078	0.196	0.058
T pRNFL	79.60 ± 17.28	74.08 ± 11.02	78.06 ± 10.08	76.80 ± 10.55
*p*-value^a^		0.708	0.438	0.447

Values are presented as the mean ± standard deviation, unless otherwise indicated.

*CSC* central serous chorioretinopathy, *G* global, *TS* superotemporal, *NS* superonasal, *N* nasal, *NI* inferonasal, *TI* inferotemporal, *T* temporal.

^a^Comparison between the baseline and values at each time point. *p-*value derived from the paired t-test.

### Factors related to final visual acuity

A correlation analysis was performed to determine the factors affecting the final visual acuity. Visual acuity at 6 months demonstrated a significant positive correlation only with visual acuity at the first visit (r = 0.313, *p* = 0.010); no significant correlation was observed between the IOP, CMT, RNFL, and GCL at the first visit and at 6 months (all *p* > 0.05).

## Discussion

In this study, we investigated the effects of acute CSCs on the inner retina using OCT. No significant difference was observed in the pRNFL and GCC thickness values when comparing the study, normal, and fellow eyes. Moreover, these values did not change significantly during the follow-up period of 6 months and were not related to the final visual acuity.

Although various causes of CSC have been suggested, studies have demonstrated retinal pigment epithelium (RPE) dysfunction and choroidal changes to be the most important factors contributing to the development of the disease^16^. Thus, most studies on CSC had focused on the outer retina, and studies on the inner retina are relatively rare. Several previous studies have demonstrated GCC to be an important indicator in various retinal diseases. A significant relationship exists between GCC and visual acuity in diabetic macular edema^8^, retinal vein occlusion^12^, and Behçet’s disease^10^; moreover, patients with retinitis pigmentosa have a reduced GCC thickness compared to healthy individuals, and this reduction is known to affect macular function^11^. Therefore, this study aimed to determine the presence of changes in the GCC in acute CSC and whether these changes could affect visual acuity.

In addition, studies have demonstrated that eyes with epiretinal membranes have a thicker pRNFL compared to normal eyes owing to traction^15,17^. However, in the case of CSC, since SRF is present in the outer retina, it is unlikely that this fluid could pass through the multiple layers of the retina and affect the GCC or pRNFL. This is supported by the fact that no significant differences were observed in the pRNFL and GCC thickness upon comparing normal and fellow eyes to CSC eyes in our study.

In the study of 30 patients with acute CSC, Nam et al.^14^ reported that the GCL-IPL thickness was lesser than the fellow eye when SRF was present, but normalized after SRF was lost, resulting in no difference from the fellow eye. In a cross-sectional study by Demirok et al.^13^ upon comparing 16 patients with acute CSC with the normal group, the mean, minimum, and superior-temporal GCL-IPL complex thickness of patients with acute CSC was significantly lesser compared to the those of the normal group. This differs from our result, which demonstrated no difference in the GCC thickness in patients with acute CSC at the first visit from fellow eyes and normal controls. However, unlike our study, which evaluated the changes over time, these studies only performed a cross-sectional analysis. Nam et al. suggested the segmentation error as one of the reasons for the thin GCL in the presence of SRF. When autosegmentation is performed using cirrus OCT equipment in the presence of SRF, the outer boundary of the RNFL or IPL cannot be accurately detected, thereby resulting in the incorrect measurement of the GCL thickness as thin. The thickness of the GCL may appear to be restored if autosegmentation is performed following SRF absorption. The authors have stated that although correcting these segmentation errors is important, it is impossible using the cirrus OCT system^14^. However, with the Spectralis OCT, manual segmentation is possible, thereby allowing correction of the segmentation errors and individual measurement of the RNFL, GCL, and IPL thickness. To minimize these measurement errors, we performed manual corrections when segmentation errors occurred owing to SRF, and did not manipulate the results in the absence of segmentation errors.

In a study of 66 patients with CSC by Lim et al.^15^ the pRNFL in the patients with CSC was thicker in the superior and temporal quadrants compared to that in the normal groups, which is attributed to the location of the leak points often in the superopnasal region of the macula in patients with CSC. The CMT of the CSC group was 515 ± 105 μm, which was thicker than the average CMT of 446.99 ± 114.18 μm at the baseline of our study. Therefore, the pRNFL measurement errors could be attributed to the inclusion of patients with presence of SRF around the optic nerve. Among the patients included in present study, the SRF did not affect the pRNFL measurements and no difference was observed in the pRNFL thickness between the study and fellow eyes during the 6-month follow-up period.

In this study, we also investigated the relationship between visual acuity and the inner retina. Inner retina, such as GCL-IPL, is one of the important determinants of visual prognosis in diabetic maculopathy or epiretinal membrane^18–20^. However, GCIPL thickness in the presence of SRF did not correlate with visual prognosis following SRF absorption in a previous study^14^. During the follow-up period of 6 months, the patients demonstrated improved visual acuity as the SRF disappeared; however, the GCC thickness and pRNFL thickness did not demonstrate any significant changes during that period. Based on these results, visual acuity of patients with CSC is presumed to be mainly affected by abnormalities in the outer retina rather than changes in the inner retina or pRNFL.

A major limitation of this study is that it was a retrospective study with a small sample size. Although manual correction was able to correct large segmentation errors, it was limited by artifacts in the OCT images of some patients. Since cirrus OCT was used in previous studies and spectralis OCT was used in this study, differences in results could exist owing to differences in the equipment. Despite these limitations, OCT segmentation errors were minimized, and the pRNFL, mRNFL, GCL, and IPL of CSC, normal, and fellow eyes were compared and the changes over time were also analyzed. Moreover, the correlation between final visual acuity, GCC, and pRNFL was also analyzed in this study.

The pRNFL and GCC did not show significant changes until 6 months after acute CSC diagnosis, and did not show significant differences upon comparison with normal and fellow eyes. Therefore, structural changes in the inner layer of the retina were not significant when acute CSC developed, and the inner layer of the retina was not related to visual acuity at 6 months after diagnosis.

## Methods

This retrospective study was conducted in accordance with the principles of the Declaration of Helsinki. The Institutional Review Board (IRB) of Hangil Eye Hospital approved this study (IRB No. 23002) and waived the requirement for informed consent from the study participants owing to the retrospective nature of the study.

### Patients

Among the patients who visited the hospital from January 2015 to April 2020, patients with acute CSC were included. Independent retinal specialists diagnosed all cases of acute CSC using fundus examinations, fluorescein angiography (FA), indocyanine green angiography (ICGA), and OCT images. A confocal scanning laser ophthalmoscope (Heidelberg Retina Angiograph, HRA; Heidelberg Engineering, Heidelberg, Germany) was utilized to conduct simultaneous FA and ICGA on all cases of CSC. The diagnosis of acute CSC was made by observing the existence of serous detachment of the neurosensory retina that affects the macula, as shown by OCT. Additionally, there was evidence of leaking at the level of the RPE on FA. The inclusion criteria were: (1) First-onset acute monocular CSC, (2) within 3 months of onset at first visit, (3) follow-up of at least 6 months, (4) SRF lasting no longer than 4 months from the onset of symptoms^2^, (5) no atrophic changes in the RPE or photoreceptors were observed during follow-up. The exclusion criteria included (1) Secondary CSC associated with systemic disease or medication history (2) the presence of other potentially conflicting retinal pathologies, such as age-related macular degeneration, polypoidal choroidal vasculopathy, pachychoroid neovasculopathy, and pachychoroid pigment epitheliopathy, (3) patients who underwent laser treatment and intravitreal injection for CSC treatment, (4) patients with glaucoma and optic nerve disease in either eye, and (5) patients who underwent retinal surgery.

Age- and gender-matched groups were created for comparison with normal eyes. The normal control group was defined as those who did not have underlying diseases, such as diabetes or hypertension; did not undergo intraocular surgery; had intraocular pressure within the normal range; and had a Snellen visual acuity of 20/20 or higher.

### Ophthalmic examination

Best-corrected visual acuity (logMAR), automatic (ARK-530A, Nidek, Gamagori, Japan) and manual corneal measurements, intraocular pressure (NT-530P, Nidek, Aichi, Japan), post-mydriatic fundus photo (Optos PLC, Dunfermline, Scotland, UK), and SD-OCT (The Spectralis HRA ± OCT Version 6.9a; Heidelberg Engineering, Heidelberg, Germany) were performed in all the patients at the first visit and at 1, 3, and 6 months.

### OCT

Macula scan was performed with 30° × 20° cube with 25 raster lines separated by 234 μm, each containing 768 pixels. Central macular thickness (CMT) was defined as the average thickness inside the 1 mm circle of the Early Treatment Diabetic Retinopathy Study (ETDRS) map. All 10 layers of the retina can be borderlined using the automatic segmentation algorithm of Heidelberg Eye Explorer software, of which the top 3 layers, RNFL, GCL and IPL, are defined as GCC. If segmentation errors occurred after automatic segmentation, the authors manually adjusted the lines representing the RNFL, GCL and IPL in each of the 25 raster lines. Manual measurements were performed by two researchers, D.I.K. and K.W.B. D.I.K performed the first manual segmentation, and based on this, K.W.B. performed the second measurement. If the results of the first measurement changed in the second measurement, researcher K.J. finally confirmed the manual segmentation measurement value.

To minimize measurement error, we did not use the central 1 mm measurement value for mRNFL, GCL, and IPL in this study. Instead, the average thickness of the 6 mm circle was calculated by dividing the volume value of the ETDRS 6 mm circle provided by OCT by 9π and then multiplying by 1000 (the average thickness of the 6 mm circle = volume of the 6 mm circle ÷ 9 π × 1000).

The pRNFL was measured by drawing a circle 3.4 mm away from the center of the disc at a scanning angle of 12°. Temporal (315°–45°), superior temporal (45°–90°), superior nasal (90°–135°), nasal (135°–225°), inferior nasal (225°–270°), and inferior temporal (270°–315°) RNFL thicknesses were assessed. The total 360° peripapillary RNFL thickness readings were averaged to obtain the global RNFL thickness.

### Statistical analysis

Statistical analysis was performed using SPSS software (version 25.0; SPSS Inc., Chicago, IL). A paired t-test was used for comparison with the fellow eye, and an independent t test was used for comparison with the normal eye. Temporal changes in the study eye were analyzed using paired t-tests. Correlation analysis with visual acuity was analyzed using the Pearson correalation analysis. A *p*-value of less than 0.05 was defined as significant.

## Data Availability

The data are not available for public access because of patient privacy concerns, but are available from the corresponding author upon reasonable request.
